# The Effect of Soy Isoflavones on Steroid Metabolism

**DOI:** 10.3389/fendo.2019.00229

**Published:** 2019-04-11

**Authors:** Amanda C. Swart, Inge D. Johannes, Thozhukat Sathyapalan, Stephen L. Atkin

**Affiliations:** ^1^Department of Biochemistry, Stellenbosch University, Stellenbosch, South Africa; ^2^Academic Diabetes, Endocrinology and Metabolism, University of Hull, Hull, United Kingdom; ^3^Research Department, Weill Cornell Medical College Qatar, Doha, Qatar

**Keywords:** soy, isoflavone, genistein, daidzein, steroid, CYP17A1, 3 β-hydroxysteroid dehydrogenase 2 (HSD3B2)

## Abstract

**Objective:** This study is a *post-hoc* analysis of steroid hormones before and after administration of pharmacological doses of soy isoflavones in a large cohort of men and women from two independent studies. Isoflavones are reported to inhibit mineralo- and glucocorticoid hormone production as well as reproductive steroids *in vivo* and *in vitro*. We focused on cytochrome P450 17α-hydroxylase (CYP17A1) which catalyses the production of dehydroepiandrosterone (DHEA), in the androgen biosynthesis pathway to elucidate effects on sex steroids *in vitro*.

**Design and Setting:** Effects of soy isoflavones on steroid levels in two studies comprising 400 patients were examined: 200 men (study 1; 3 months duration) and 200 postmenopausal women (study 2; 6 months duration), randomized to consume 15 g soy protein with 66 mg isoflavones (SPI) or 15 g soy protein alone without isoflavones (SP) daily. Effects of genistein and daidzein on steroid metabolism were determined *in vitro*, in HEK293 cells expressing CYP17A1 and in the human adrenocortical carcinoma H295R cell model.

**Results:** SPI decreased serum dehydroepiandrosterone sulfate (DHEAS) levels in both men and women (*P* < 0.01), with decreased androstenedione (A4) (*P* < 0.01) in women not observed in men (*P* < 0.86). Cortisol, cortisone, 11-deoxycortisol, aldosterone, testosterone (T), or estradiol (E2) levels were unchanged. The dual hydroxylase and lyase activity of CYP17A1, which catalyses the biosynthesis of androgen precursors, and 3β-hydroxysteroid dehydrogenase (3βHSD2) were investigated *in vitro*. In transiently transfected HEK293 cells, only the lyase activity was inhibited by both genistein, 20% (*P* < 0.001) and daidzein, 58% (*P* < 0.0001). In forskolin-stimulated H295R cells DHEA production was decreased by daidzein (*P* < 0.05) and genistein, confirming inhibition of the lyase activity by the isoflavones.

**Conclusion:** *In Vivo* clinical data suggested inhibition of CYP17A1 17,20 lyase within the adrenal in men and within the ovary and adrenal in females. This was confirmed *in vitro* with inhibition of the lyase activity by both genistein and daidzein. In addition, 3βHSD2 was inhibited perhaps accounting for decreased A4 levels observed in females. The decreased DHEAS and A4 levels together with the inhibition of the 17,20 lyase activity of CYP17A1, may impact production of androgens in clinical conditions associated with androgen excess.

ISRCTN number: ISRCTN55827330

ISRCTN number: ISRCTN 90604927

## Introduction

Dietary intake of isoflavones in Asian diets has been estimated to be in the range of 30–50 mg/day of combined isoflavone aglycone equivalents that are mainly genistein and daidzein (1, 2). Health effects of soy consumption have been reported for protection against breast and prostate cancer (3–5), osteoporosis (6), cardiovascular diseases (7, 8), and the alleviation of hot flashes (9). This has led to the development of supplements containing isoflavones and the fortification of foods with soy isoflavones (10, 11). However, there are concerns that soy may adversely affect thyroid function in susceptible individuals (12–14).

We have shown that high-dose isoflavone intake, in comparison with isoflavone-free soy, impairs thyroid function in patients with type 2 diabetes (T2DM) (Study 1) (15) and also in post-menopausal healthy women (Study 2) (16). Study 1 was a randomized double-blind parallel study investigating the effect of soy isoflavones on testosterone (T) serum concentrations in men with T2DM; whereas study 2 was a double-blind randomized parallel study investigating the effect of high dose isoflavone intake on bone turnover markers in women within 2 years of onset of menopause. The effect of soy isoflavones on other steroid hormones, is not well-established.

**Figure 1:**
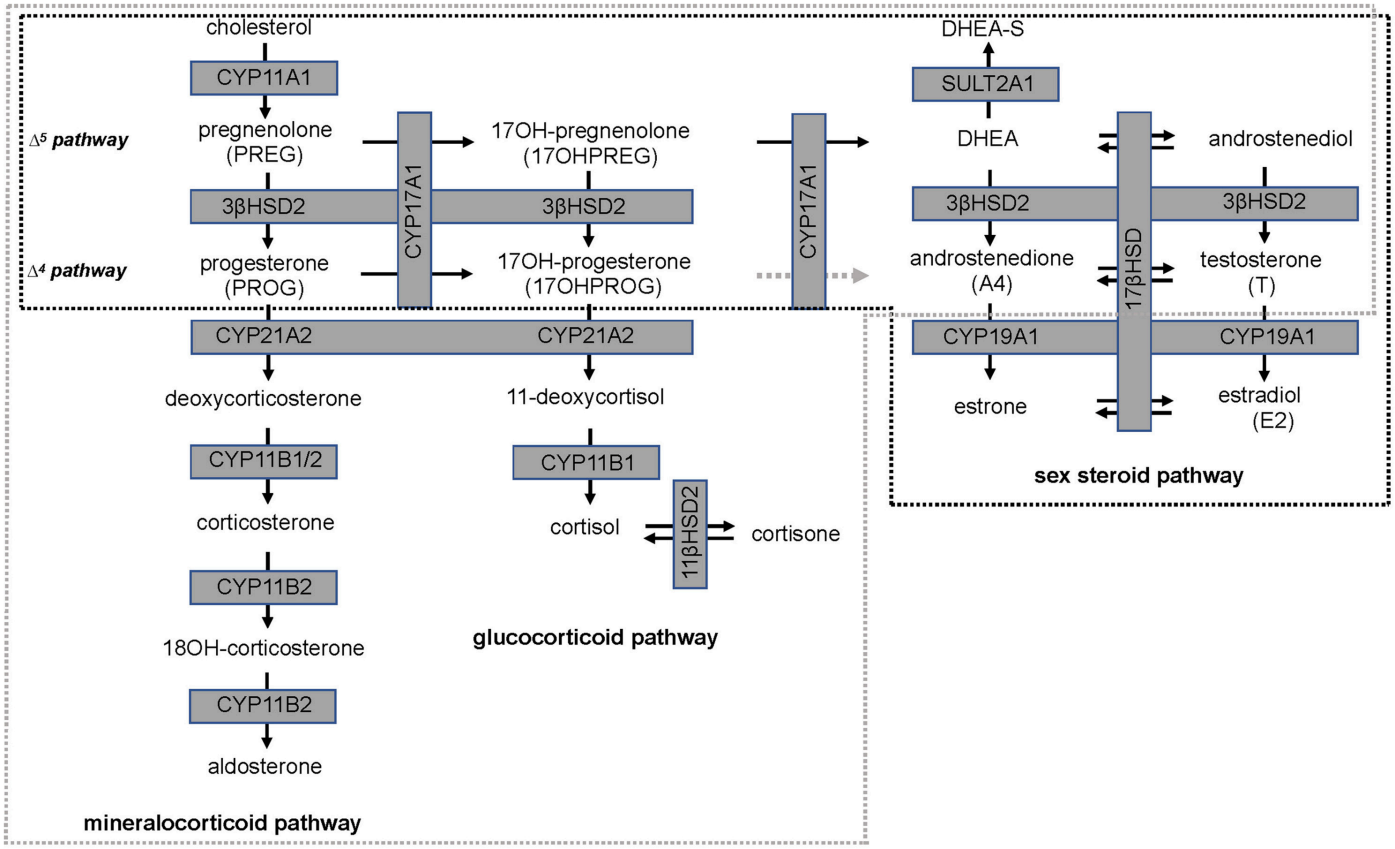
Steroid hormone biosynthesis in the mineralocorticoid, glucocorticoid, and sex steroid pathways. Production is catalyzed by steroidogenic enzymes (boxed, shaded in gray) in the adrenal (broken gray line) and in steroidogenic tissue (broken black line). The conversion of 17-hydroxyprogesterone (17OHPROG) to androstenedione (A4) by CYP17A1 in humans is negligible (broken gray arrow).

Isoflavones have been reported to have an inhibitory action on steroid pathways potentially impacting the biosynthesis of estradiol (E2) and T, although this has not been observed clinically. A study which undertook a meta-analysis of clinical outcomes of effects of isoflavones on T, free T, and sex-hormone binding globulin (SHBG), reported that neither isoflavones nor soy protein effected these three parameters significantly (17). However, 3β-hydroxysteroid dehydrogenase type 2 (3βHSD2), which catalyses the production of sex steroid precursors, has been reported to be inhibited by isoflavones, together with cytochrome P450 21 hydroxylase (CYP21A2). CYP21A2 catalyses the production of steroid hormone intermediates in the adrenal (18, 19) thus potentially impacting steroidogenesis (20, 21). A schematic of the relevant steroid pathways is shown in Figure 1 in which cytochrome P450 side chain cleavage (CYP11A1) catalyses the conversion of cholesterol, the common precursor to all steroid hormones, to pregnenolone (PREG). PREG is subsequently channeled into the androgen pathway (Δ^5^ pathway) by cytochrome P450 17α-hydroxylase (CYP17A1) via 17-hydroxypregnenolone (17OHPREG) or into the mineralocorticoid pathway by 3βHSD2. In this pathway, the 3βHSD2 product, progesterone (PROG), is converted by CYP21A2 and aldosterone synthase (CYP11B2) via the intermediates, deoxycorticosterone and corticosterone, to aldosterone. 17OHPREG is also converted by 3βHSD2 to form 17OHPROG, which is ultimately converted to cortisol by CYP21A2 and cytochrome P450 11β-hydroxylase (CYP11B1). 17OHPROG is also a product of the hydroxylase activity of CYP17A1 in the conversion of PROG (Δ^4^ pathway). In humans, the conversion of 17OHPROG to androstenedione (A4) is negligible with the latter produced from dehydroepiandrosterone (DHEA) via the Δ^5^ pathway via 3βHSD2 (22). In the adrenal, DHEA is predominantly converted to dehydroepiandrosterone sulfate (DHEAS), which is the most abundant steroid produced at levels 30-fold higher than DHEA, with the latter increasing ~50-fold upon adrenocorticotropic hormone (ACTH) stimulation (23).

A number of studies have reported that isoflavones inhibit mineralo- and glucocorticoid hormone production as well as reproductive steroids *in vivo* and *in vitro* (18–21). The present study was therefore initiated to do a *post-hoc* analysis of steroid hormones before and after administration of pharmacological doses of soy isoflavones in a large cohort of men and women from two independent studies. In order to elucidate the effects of two isoflavone glycones, daidzein, and genistein, on steroid pathways *in vitro*, we focused on CYP17A1 since the enzyme catalyses the production of DHEA, the precursor to sex steroids in steroidogenic tissue and to DHEAS in the adrenal. Firstly, we investigated the CYP17A1 hydroxylase activity by assaying the conversion of PROG and the CYP17A1 17,20 lyase activity by assaying the conversion of 17OHPREG in the presence of isoflavones in transiently transfected HEK293 cells, void of interfering steroidogenic enzymes. This was followed by further investigations into effects on the lyase activity in H295R cells, a human adrenal cell model. These cells produce all the steroids characteristic of the cortical zones which include the mineralocorticoids, glucocorticoids and adrenal androgen precursors. In addition, due to the expression of low levels of 17β-hydroxysteroid dehydrogenase (17βHSD) and cytochrome P450 aromatase (CYP19A1), these cells produce T, estrone and E2, albeit at low levels. Since H295R cells are insensitive to ACTH, forskolin was used to stimulate steroidogenesis and thus increase the steroid flux toward DHEA. Forskolin, a diterpene, mimics the actions of ACTH via the activation of adenyl cyclase pathways in adrenal cells (24). In order to eliminate downstream conversion of the steroids in the Δ^5^ pathway, trilostane, a 3βHSD2 inhibitor was added, blocking the conversion of PREG and 17OHPREG in aldosterone and cortisol production respectively. The effects of genistein and daidzein on DHEA production could therefore be investigated under basal and stimulated conditions together with the addition of 17OHPREG to analyse the lyase activity of CYP17A1.

## Materials and Methods

### *In vivo* Effects of Isoflavones on Hormonal Parameters

#### Patients and Protocols

**Figure 2:**
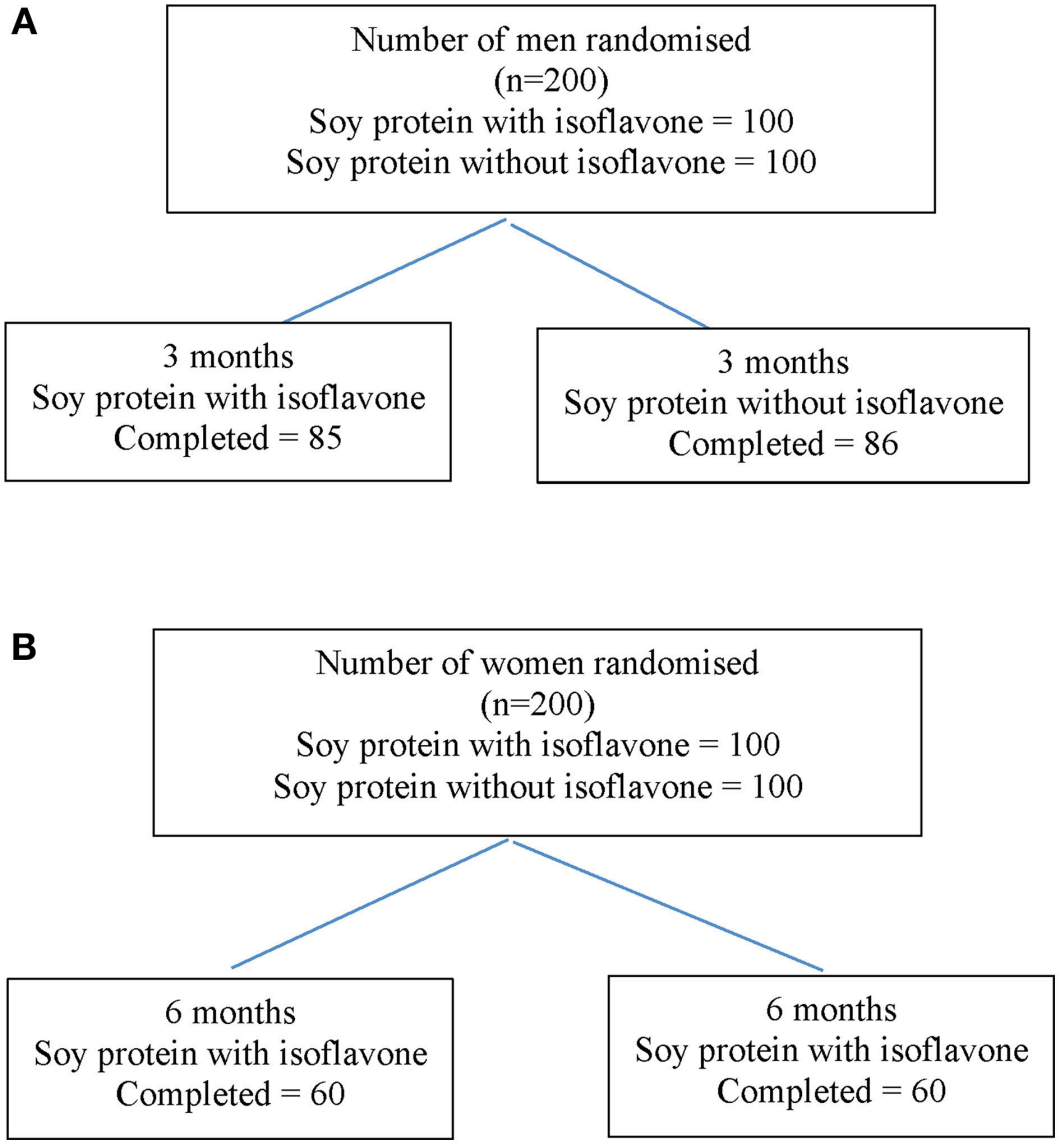
Flow diagram of participant details from recruitment to the end of study 1 **(A)** and study 2 **(B)**.

Study 1 involved 200 men aged between 45 to 75 years with T2DM, low early morning total T concentrations (total T less than the lower level of the reference range of 12 nmol/L) and normal gonadotropins who participated in a randomized double-blind parallel study investigating the effect of soy isoflavones on serum T concentrations. They were randomized either to consume 15 g soy protein with 66 mg of isoflavones (SPI) per day or 15 g soy protein alone without any isoflavones (SP) per day for 3 months in the form of snack bars (Figure 2A). Study 2 involved 200 healthy women within 2 years of the onset of menopause who were recruited (16) to assess the impact of high dose soy isoflavones on bone turnover markers (Figure 2B). Both studies received ethical approval by the Research Ethics Committee (East Yorkshire & North Lincolnshire Research Ethics Committee, ref: 09/H1304/45 and 09/1304/45). Computer-generated block randomization lists were performed by Essential Nutrition Ltd, Brough, UK, who held the randomization codes for both studies.

The intervention consisted of a snack bar containing 7.5 g isolated soy protein powder (Solcon F, Solbar Industries, Israel) with 33 mg of isoflavones (SPI) (Solgen 40, Solbar Industries, Israel) given twice daily between meals (15 g soy protein & 66 mg of isoflavones per day), or 7.5 g of the isolated soy protein given twice daily (15 g soy protein per day without isoflavones per day) as control (SP). The latter had an isoflavone concentration of less than 300 parts per billion following serial alcohol extraction by Dishman Ltd, India (16). The product isoflavones were assayed by FERA, Sand Hutton, UK (16). Analysis showed the composition of the dose materials to be 12% glycitein, 35% daidzein, and 54% genistein as aglycones. Ninety percent of the isoflavones were in the primary glucoside form, with the remaining 10% as aglycones, specifically malonyl and acetyl glucosides. The snack bars were consumed twice daily between meals (3 months for study 1 and 6 months for study 2). The study bars were prepared and packaged by Halo foods, Swindon, UK. Soy bars were identical and had similar macronutrient content and a tasting panel had determined that there was no discernible difference between the two products.

Steroid hormone levels were quantified using liquid chromatography tandem-mass spectrometry (LC-MS/MS) (25). The isoflavones were extracted and analyzed from serum by LGC, Fordham, Cambridgeshire, UK using isotope-dilution LC-MS/MS (26). LC-MS/MS was conducted using a Sciex 4000 QTRAP with separation achieved using a C_18_ column with the mobile phase consisting of acetonitrile and water, both containing acetic acid (15).

### *In vitro* Influence of Daidzein and Genistein on Steroid Hormones

#### Materials

Pregnenolone (PREG), progesterone (PROG), 17-hydroxypregnenolone (17OHPREG), 17-hydroxyprogesterone (17OHPROG), androstenedione (A4), and dehydroepiandrostenedione (DHEA) were purchased from Steraloids (Newport, USA). Deuterated steroids were acquired from Cambridge isotopes (Andover, USA) and included testosterone 1,2-D2 (D2-T; 98%), progesterone 2,2,4,6,6,17,21,21,21-D9 (D9-P4; 98%), and 4-androstene-3,17-dione 2,2,4,6,6,16,16-D7, (D7-A4, 97%,). HEK293 cells were purchased from American Type Culture Collection (Manassas, USA) and H295R cells were a kind gift from Prof. WE. Rainey, Ann Arbor, University of Michigan. The CYP17A1/pcDNA3.1 plasmid construct was gifted by Prof CE Flück (University of Bern, Switzerland). NucleoBond® Xtra Maxi plasmid DNA isolation kits were purchased from Macherey-Nagel (Düren, Germany). Corning® CellBIND® Surface 12- and 24-well tissue culture plates were acquired from Corning® Life Sciences (NY, USA) whilst XtremeGene HP^®^ DNA transfection reagent was purchased from Roche Diagnostics (Manheim, Germany). Penicillin-streptomycin, fetal bovine serum (FBS) and trypsin-EDTA were supplied by Thermo Fisher Scientific (Waltham, USA). Methyl tert-butyl ether (MTBE), analytical-grade methanol and formic acid were purchased from Sigma-Aldrich (St. Louis, MA, USA). Dulbecco's modified Eagle's Medium (DMEM), DMEM/F12 and gentamicin were purchased from Invitrogen/Gibco (Grand Island, New York, USA). Cosmic calf serum (CCS) was supplied by HyClone®, Thermo Scientific (South Logan, Utah, USA). The Acquity UPC^2^ BEH C_18_ column was obtained from Waters Corporation (Milford, USA). FOODFRESH CO_2_ was supplied by Afrox (Cape Town, South Africa). Daidzein and genistein were purchased from Sigma-Aldrich (St. Louis, MA, USA). All other chemical reagents were of the highest quality and supplied by reputable scientific distributors.

#### Methods

Cells were cultured in a stable environment at 37°C, 5% CO_2_ and 90% relative humidity. HEK239 cells were cultured in DMEM supplemented with 10% FBS and 1% penicillin-streptomycin; H295R cells in DMEM/F_12_, supplemented with L-glutamine, 15 mM HEPES, pyridoxine, 1.125 g NaHCO_3_/L, 1% penicillin streptomycin, 0.01% gentamicin, and 10% CCS. Cells were verified to be mycoplasm free and cell viability not compromised by the addition of the isoflavones (10 μM). Experimental procedures were carried out once cells were 80% confluent and had reached a minimum of three to five passages.

Confluent HEK293 cells were seeded into 24 well plates at a density of 2 × 10^5^ cells/mL, 0.5 mL/well, and transiently transfected after 24 h with 0.5 μg CYP17A1/pcDNA3.1 or pCIneo (negative control without DNA insert) per well according to manufacturer's instructions. Cells were incubated for 48 h after which media was replaced with experimental media (culture media containing 0.1% FBS) to which steroid substrates (PROG and 17OHPREG) were added with and without 10 μM genistein or daidzein. Steroid conversion was assayed by collecting media aliquots (500 μL) at specific time intervals followed by steroid extraction (27).

Confluent H295R cells were seeded into 12 well plates (4 × 10^5^ cells/mL, 1 mL/well) and incubated for 48 h after which media was replaced with experimental media (growth medium containing 0.1% CCS) with and without 10 μM forskolin and 10 μM trilostane. Cells were incubated for a further 12 h and media replaced with experimental media (i) with and without isoflavones (10 μM) to assay CYP17A1 activity under basal conditions and (ii) containing 1 μM 17OHPREG with and without isoflavones (10 μM) to assay lyase activity in forskolin-stimulated cells in which inhibition of 3βHSD2 activity by trilostane prohibited downstream steroid conversion. Steroid metabolism was assayed after 48 h by collecting media aliquots (500 μL) and extracting steroid metabolites.

Steroids were extracted by liquid/liquid extraction as previously described (27). Briefly, organic solvent extraction was carried out using a ratio of 1:3 (v/v) media to MTBE after the addition of 100 μL internal standard mix consisting of D9-P4, D7-A4, D2-T (1.5 ng each), prior to extraction. Samples and standards were vortexed at 800 RPM for 5 min and incubated at −80°C for an hour. The liquid organic phase was transferred to test tubes and dried under nitrogen at 55°C. The dried residue (samples and standards) were resuspended in 150 μL 50% methanol and analyzed using Ultra-Performance convergence chromatography-tandem mass spectrometry (UPC^2^-MS/MS).

The steroid metabolites were separated using an Acquity UPC^2^ BEH C_18_ column (2.1 × 100 mm, 1.7 μm particle size) with an Acquity UPC^2^ system (Waters Corporation, Milford, USA). Quantitative mass spectrometric detection was carried out using a Xevo TQ-S triple quadrupole mass spectrometer (Waters, Milford, USA). The mobile phase consisted of liquid CO_2_ modified with methanol. A 2.5 min linear gradient from 4 to 25% methanol was used to separate the steroids using a constant flow rate of 2.0 mL/min.

#### Statistical Analysis

This was a *post-hoc* exploratory analysis of two *in vivo* studies to understand the impact of high dose isoflavones on steroid concentrations and hence *a priori* power analyses were not done for changes in steroid concentrations.

Baseline continuously distributed data is presented as median (25/75th centiles); categorical data by n (%). Within-group differences (difference between 12 week/24 week values and baseline values) are shown for each treatment group separately by a mean and a standard deviation (SD). Between-group comparisons were performed using the independent sample *t*-test. The *t*-test assumes equal variance between groups. For all statistical analyses, a two-tailed *P* < 0.05 was considered to indicate statistical significance. Statistical analyses were performed using the STATA statistical computer package (StataCorp 2013. *Stata Statistical Software:* StataCorp LP, USA).

GraphPad Prism (version 7.0) software (GraphPad Software, San Diego, California) was used for statistical analysis of *in vitro* data. Experiments were carried out in triplicate with results depicting the mean value ± SEM, with 3 independent experiments conducted, or mean value ± SD. Statistical significance was determined using a two-way ANOVA followed by a Dunnett's multiple comparison test. Probability (*P*) values lower than 0.05 were considered statistically significant.

## Results

### Analysis of *in vivo* Effects on Hormonal Parameters

The demographics for study 1 (men; median (25/75th centiles); BMI and age control 31.6 (29.2, 35.0) and 52.0 years (50.0, 55.0), BMI and age active preparation 31.8 (28.8, 34.7) and 52.0 years (50.0, 55.0). Study 2 (median (25/75th centiles); BMI and age control 24.6 (22.7, 28.4) and 52.0 years (50.0, 55.0), BMI and age active preparation 26.3 (24.3, 30.7) and 52 years (49, 56). In Study 1, there was a significant decrease in DHEAS with SPI supplementation (Table 1, *P* < 0.01) whereas in study 2 there was a significant decrease in both DHEAS and A4 with SPI supplementation (Table 2, *P* < 0.01). No changes were observed in basal levels of T, E2, cortisol, cortisone, A4 (men only), 17OHPROG or aldosterone (Tables 1, 2). No stimulation tests for adrenal function were undertaken.

**Table 1:** Comparison of hormonal parameters levels with active (66 mg isoflavones) and control (0 mg isoflavones) preparations in 171 men treated for 12 weeks.

	**Active SPI** ***n*** **=** **85**		**Control SP** ***n*** **=** **86**		**Difference**		
**Parameter**	**Baseline**	**12 weeks**	**Baseline**	**12 weeks**	**Active**	**Control**	**(95% CI) *p*-value**
Total testosterone (nmol/L)	9.83 ± 0.21	11.34 ± 0.36	9.22 ± 0.23	10.33 ± 0.30	1.51 ± 0.25	1.10 ± 0.27	(−0.31–1.14) 0.26
% free testosterone	2.66 ± 0.56	2.63 ±0.54	2.73 ± 0.63	2.78	0.03 ± 0.04	0.05 ± 0.07	0.22
Absolute free testosterone (nmol/L)	0.21 ± 0.05	0.24 ± 0.06	0.20 ± 0.05	0.23 ± 0.06	0.03 ± 0.06	0.03 ± 0.05	0.80
Androstenedione (nmol/L)	2.1 ± 0.09	2.3 ± 0.1	2.0 ± 0.09	2.1 ± 0.9	−0.12 ± 0.08	−0.05 ± 0.9	(−0.15–0.31) 0.52
17hydroxyprogesterone (nmol/L)	2.1 ± 0.13	2.2 ± 0.11	2.0 ± 0.09	2.0 ± 0.09	−0.08 ± 0.11	−0.06 ± 0.07	(−0.25–0.30) 0.86
SHBG (nmol/L)	32.32 ± 1.38	32.01 ± 1.53	31.65 ± 1.50	30.26 ± 1.40	−0.30 ± 0.71	−1.34 ± 0.59	(0.81–2.89) 0.27
FSH (iu/L)	7.89 ± 0.70	8.16 ± 0.71	7.27 ± 0.50	7.33 ± 0.53	0.27 ± 0.17	0.07 ± 0.12	(−0.21–0.61) 0.34
LH (iu/L)	4.42 ± 0.32	4.76 ± 0.31	4.26 ± 0.25	4.43 ± 0.28	0.34 ± 0.12	0.16 ± 0.15	(−0.20–0.55) 0.35
Estradiol (pmol/L)	85.01 ± 2.94	87.3 ± 2.83	88.91 ± 2.69	89.32 ± 2.84	2.29 ± 2.64	0.41 ± 2.43	(−5.24–9.0) 0.60
DHEA (nmol/L)	4.0 ± 0.4	4.1 ± 0.4	3.3 ± 0.3	3.3 ± 0.2	−0.11 ± 0.22	0.06 ± 0.23	(−0.49–0.82) 0.6
DHEAS (umol/L)	2.93 ± 0.20	2.76 ± 0.17	2.69 ± 0.18	2.87 ± 0.19	−0.26 ± 0.07	0.18 ± 0.07	(−0.57–0.16) 0.01
Cortisol (nmol/L)	360 ± 11	375 ± 12	363 ± 13	392 ± 12	−12.7 ± 12.2	−28.0 ± 12.6	(−50.1–19.4) 0.38
Cortisone (nmol/L)	55.9 ± 1.2	58.1 ± 1.3	55.1 ± 1.2	75.7 ± 1.2	2.1 ± 1.1	2.2 ± 1.1	(−3.1–2.8) 0.9
Aldosterone (pmol/L)	108 ± 8	134 ± 10	147 ± 16	156 ± 20	27 ± 11	12 ± 14	(−21–53) 0.4

*FSH, follicle stimulating hormone; LH, luteinising hormone; DHEAS, dehydroepiandrostenedione-sulfate; SHBG, sex hormone binding globulin*.

**Table 2:** Comparison of hormonal parameters levels with active (66 mg isoflavones) and control (0 mg isoflavones) preparations in 120 women treated for 26 weeks.

	**Active**		**Control**		**Difference**		
**Parameter**	**Baseline**	**26 weeks**	**Baseline**	**26 weeks**	**Active**	**Control**	**(95% CI) *p*-value**
Total testosterone (nmol/L)	0.71 (0.3)	0.67 (0.27)	0.68 (0.32)	0.66 (0.3)	0	0.02	−0.02 (−0.09,0.03) 0.39
Androstenedione (nmol/L)	4.1 (2.1)	2.2 (1.5)	3.8 (2.8)	3.4 (2.9)	−1.9	−0.2	−2.0 (−2.8, −1.2) < 0.01
17hydroxyprogesterone (nmol/L)	2.0 ± 0.09	2.1 ± 0.19	2.1 ± 0.11	2.1 ± 0.06	−0.09 ± 0.10	−0.02 ± 0.05	(−0.26–0.32) 0.92
SHBG (nmol/L)	56.5 (25)	52.7 (24.3)	56.6 (26.2)	53 (22.2)	−3.8	−3.4	−0.4 (−5.5,4.6) 0.86
FSH (iu/L)	77.5 (27.9)	79.8 (34.4)	70.3 (31)	73.8 (33.3)	2.4	3.2	−6 (−15.2,3.1) 0.19
LH (iu/L)	34.1 (12.4)	35.5 (13.1)	33.1 (14.2)	35.3 (14.6)	1.2	3	−2.8 (−6.8,1.1) 0.16
Estradiol (pmol/L)	78.1 (32.9)	97.9 (89.7)	109.9 (113)	111.8 (122)	22	−4.3	26.4 (−19.5,2.4) 0.25
DHEAS (umol/L)	2.8 (1.5)	1.4 (1.3)	2.6 (2.1)	2.5 (1.3)	−1.3	−0.22	−1.16(−1.77, −0.95) < 0.01
Cortisol (nmol/L)	331	345	365	349	21	16	(−37–45) 0.84
Cortisone (nmol/L)	56.4	55.9	58.3	56.7	1.4	2.0	(−22.5–79.1) 0.27
11 deoxycortisol (nmol/L)	0.5 ± 0.04	0.57 ± 0.06	0.70 ± 0.11	0.75 ± 0.13	0.07	0.05	(−0.25–0.2) 0.81
Aldosterone (pmol/L)	157 ± 13	171 ± 16	165 ± 18	176 ± 20	14	11	(−38–39) 0.98

*FSH, follicle stimulating hormone; LH, luteinising hormone; DHEAS,dehydroepiandrostenedione-sulfate; SHBG, sex hormone binding globulin*.

### Analysis of *in vitro* Effects on Steroid Hormone Production

The influence of the isoflavones on CYP17A1 activity was ascertained in transiently transfected HEK293 cells and in the H295R adrenal cell model. Although pharmacological doses of isoflavones of 66 mg per day resulted in circulating levels of 127 and 70 nmol/L daidzein and 434 and 338 nmol/L genistein in males and females respectively, uptake by target tissue remains unknown as does accumulation and elimination of isoflavones. Previous reports showed significant inhibition of both 3βHSD2 and CYP21A2 at 12.5 μM; and of 3βHSD2 at lower concentrations of 1 μM genistein and 3.1 μM daidzein (18). Since these concentrations would potentially modulate steroidogenesis in both the adrenal and steroidogenic tissue, we conducted our *in vitro* assays with 1 and 10 μM genistein and daidzein.

**Figure 3:**
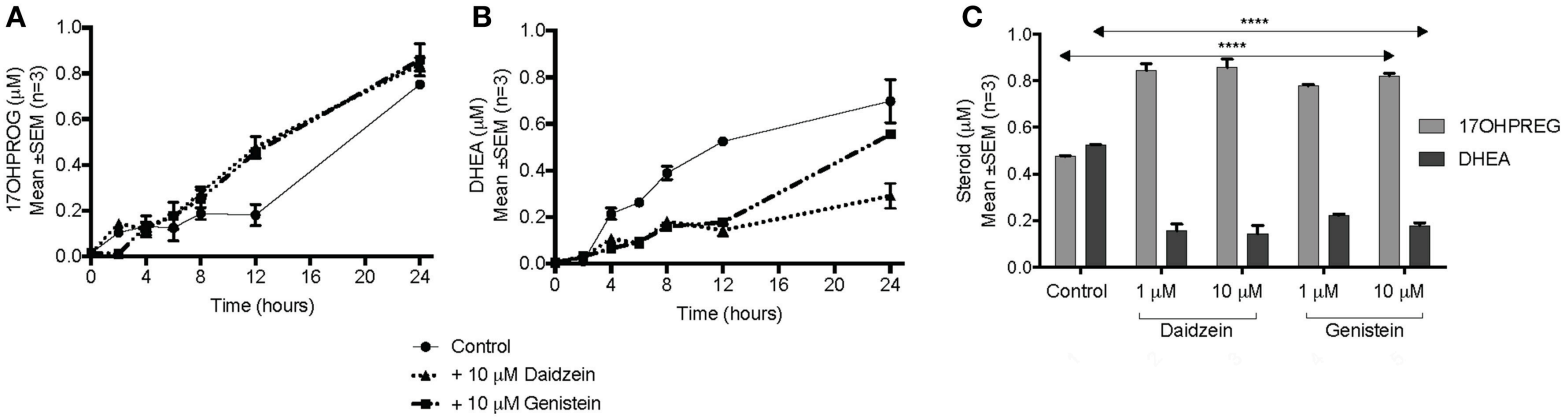
Assay of CYP17A1 hydroxylase and lyase activity in transiently transfected HEK293 cells in the presence of isoflavones. **(A)** conversion of 1 μM PROG to 17OHPROG; **(B)** and **(C)** conversion of 1 μM 17OHPREG to DHEA with substrate only (solid line) and in the presence of 10 μM daidzein (^**…….…**^) and 10 μM genistein (–. –.). Data was analyzed by a two-way ANOVA, followed by a unpaired *t*-test. Results are expressed as the mean ± SEM (*n* = 3, ****P < 0.0001).

The hydroxylase activity was firstly assayed in the presence of the isoflavones using PROG as substrate since CYP17A1 does not readily convert the hydroxylated intermediate to A4 in humans. In HEK293 cells transiently transfected with CYP17A1, without and in the presence of daidzein and genistein, the conversion of PROG to 17OHPROG (Figure 3A) showed that neither isoflavone inhibited the hydroxylase activity with ~0.2 μM substrate remaining after 24 h. Neither of the isoflavones inhibited the conversion of PREG to 17OHPREG (data not shown). In order to assay the 17,20 lyase activity in the Δ^5^ pathway, the conversion of 17OHPREG to DHEA (Figure 3B) was assayed. Both isoflavones inhibited the lyase activity significantly with daidzein exhibiting a greater inhibitory effect. While ~70% 17OHPREG was converted to DHEA after 24 h in the absence of isoflavones, only ~33% was converted to DHEA in the presence of daidzein (*P* < 0.0001) and ~55% in the presence of genistein (*P* < 0.001). The assay was also conducted at lower concentrations (1 μM) of isoflavones which also resulted in significant inhibition of the lyase activity (Figure 3C).

**Figure 4:**
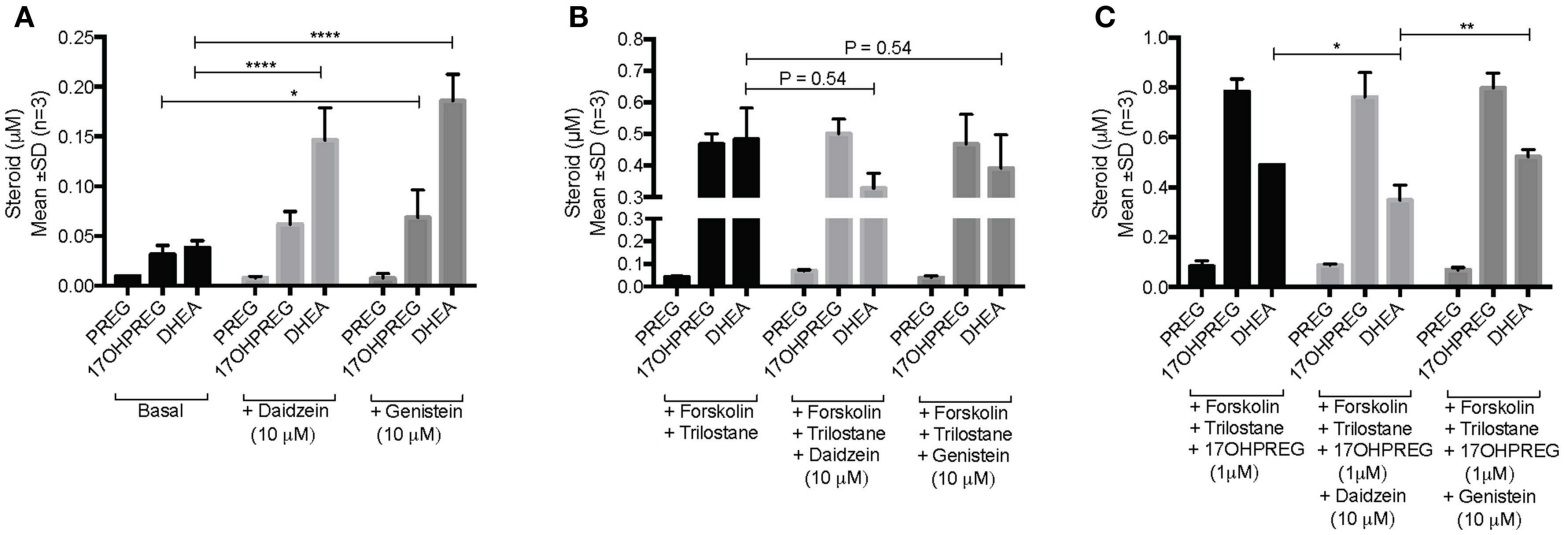
Assay of CYP17A1 hydroxylase and lyase activity in the Δ^5^ pathway in H295R cells in the presence of 10 μM daidzein and 10 μM genistein after 48 h. **(A)** basal endogenous steroid levels without and in the presence of isoflavones; **(B)** endogenous steroid levels in forskolin-stimulated cells in the presence of trilostane, a 3βHSD inhibitor, without and in the presence of isoflavones; and **(C)** the addition of 1 μM 17OHPREG to forskolin-stimulated cells, in the presence of trilostane, without and in the presence of isoflavones. Data was analyzed by a two-way ANOVA, followed by a Dunnett's multiple comparison test. Results are expressed as the mean ± SD (*n* = 3, *P < 0.05, **P < 0.01, ****P < 0.0001).

The influence of daidzein and genistein on the Δ^5^ pathway was subsequently investigated in the adrenal H295R cell model. Basal endogenous steroid levels are depicted in Figure 4A showing the addition of isoflavones resulting in 17OHPREG and DHEA levels increasing significantly by ~3.8- and ~4.85-fold, respectively. Basal levels were ~0.035 μM with 17OHPREG increasing to 0.06 μM and DHEA to 0.14 and 0.19 μM in the presence of daidzein and genistein, respectively. Treatment with forskolin and trilostane (Figure 4B) increased basal levels ~12-fold with 17OHPREG and DHEA levels increased to ~0.47 μM. The addition of daidzein and genistein did not change 17OHPREG levels; however, DHEA levels were decreased, approaching significance. We subsequently assayed the conversion of 1 μM 17OHPREG under these conditions (Figure 4C). After 48 h, 17OHPREG and DHEA were detected at 0.78 and 0.5 μM respectively, and in the presence of trilostane and forskolin, 17OHPREG remained unchanged with only daidzein significantly lowering DHEA levels to 0.34 μM.

## Discussion

The data show that dietary SPI supplementation modulated the androgen biosynthesis pathway *in vivo* showing that in both men and women DHEAS levels were significantly decreased by the SPI preparation compared to soy alone, suggesting that the isoflavones may impact either on the steroidogenic Δ^5^ pathway or the sulphation/desulfation of DHEA. Neither daidzein nor genistein influenced levels of mineralocorticoids or glucocorticoids significantly in the study subjects. Aldosterone levels did not change significantly while cortisol levels remained comparable together with the precursor steroids in both study groups remaining unchanged. The inactivation of cortisol was also not influenced with cortisol:cortisone ratios unchanged after consumption of isoflavones (6.4 in women and 6 in men). Corroborating previous reports, (17) T, free T and SHBG levels were also not influenced by the isoflavones, suggesting that the influence of the isoflavones on these steroids is not clinically significant. However, A4 levels were significantly decreased in women consuming isoflavones. In women, A4 is the product of DHEA and the precursor steroid of T, both products of the ovary and adrenal (Figure 1). As mentioned previously, the adrenal is the primary site of DHEAS biosynthesis in men and women and most of the DHEA produced is converted to DHEAS. A4 may also be biosynthesized in the desulfation of DHEAS via the subsequent conversion of DHEA to A4, or *de novo* from cholesterol in both the adrenal and steroidogenic tissue such as the ovary and gonads. It was therefore essential to elucidate the effect isoflavones have on the androgen biosynthesis pathway *in vitro*.

In sex steroid biosynthesis pathway PREG is channeled toward DHEA via 17OHPREG, catalyzed by CYP17A1, prompting us to investigate the influence of the isoflavones on CYP17A1 as the enzyme is at the branchpoint of adrenal steroidogenesis catalyzing the production of mineralocorticoids, glucocorticoids, and sex steroids. Inhibition of the hydroxylase and lyase activity of CYP17A1 would channel steroid metabolites into the mineralocorticoid pathway. Inhibition of the dual activity, would increase the flux toward mineralocorticoid production as has been shown with the enzyme's inhibition by abiraterone in the treatment of prostate cancer resulting in not only suppressed androgen production, but also hypertension and hypokalemia (28). Inhibition of the lyase activity only, would result in a decrease in the biosynthesis of androgens, channeling the steroid flux toward the mineralo- and glucocorticoid pathways. In the adrenal as well as in steroidogenic tissue CYP17A1 catalyses the 17α-hydroxylase of PROG and PREG to their hydroxyl intermediates. The subsequent 17,20 lyase of 17OHPREG only yields DHEA in the Δ^5^ pathway. Data clearly show that while the hydroxylase activity was not affected (Figure 3A) the lyase activity was inhibited significantly (Figure 3B). Previous studies had reported that neither daidzein nor genistein inhibited CYP17A1 (19). This study however, did not investigate the hydroxylase and 17,20 lyase activities as individual reactions.

The inhibition of the lyase activity of CYP17 by the isoflavones was investigated further in the H295R adrenal model to determine effects on the Δ^5^ pathway in the presence of competing enzymes viz 3βHSD2, SULT2A1, and 17βHSD. In the presence of both isoflavones, 17OHPREG and DHEA levels increased indicating the inhibition of 3βHSD2 activity, impacting steroid metabolism in the mineralocorticoid, glucocorticoid, and androgen pathways. Both daidzein and genistein have been shown to be competitive inhibitors of 3βHSD2 with daidzein shown to be a competitive inhibitor of CYP21A2 as well (19), which would lead to a decrease in downstream metabolites in the three pathways as has also been reported by other groups (29–31). Subsequent to these studies, it was shown that the presence of 10 μM daidzein and genistein in H295R cells stimulated with forskolin to mimic the action of ACTH, increased PREG and DHEA levels significantly, corroborated by our study. The authors attributed these changes, together with the A4 and 17OHPROG levels decreasing significantly, to the inhibition of 3βHSD2 only (32). It is possible that the increased 17OHPREG and DHEA levels could be due to the inhibition of 3βHSD2 and CYP21A2 increasing these precursor steroids being shunted in the Δ^5^ pathway as is shown in our data. On the other hand, higher DHEA levels may also be due to the inhibition of 17βHSD5 which catalyses the further conversion of DHEA to androstenediol in the adrenal. Inhibition of the reductive activity of the enzyme by the isoflavones has been reported with DHEA as substrate –daidzein exhibited an IC_50_ at 10 μM and genistein IC_50_ at 1 μM (33). With DHEA being converted predominantly to DHEAS in the adrenal, inhibition of sulfotransferase (SULT2A1) activity may thus also be contributing to DHEA levels. Such inhibition would result in lower DHEAS levels which may account for our *in vivo* findings. However, it has been reported that neither daidzein nor genistein inhibit SULT2A1 at levels as high as 25 μM (34). The decreased DHEAS levels detected in our study in both women and men would probably not be due to the inhibition of adrenal production of DHEAS, but rather the modulation of the steroid shunt in the Δ^5^ pathway. Since the adrenal is an important source of androgen production in females, the inhibitory effect of the isoflavones on the lyase activity of CYP17A1 in the pathway and subsequent inhibition of 3βHSD2, may account for the decreased A4 levels in women. It is also interesting to note that in women the decreased DHEAS levels were 2-fold lower than those of the control group and, while A4 levels in males were not affected by the isoflavones, they were comparable to the suppressed A4 levels detected in women after 26 weeks.

The modulation of DHEAS levels by isoflavones is complex and analysis of the mechanism/s impacting the biosynthesis and the metabolism of the conjugated steroid are not fully resolved. Potentially contributing to DHEAS levels is the availability of the DHEA precursor steroid in the Δ^5^ pathway determined by the activity of CYP17A1 and by the subsequent metabolism of DHEA by 17βHSD and 3βHSD2. *In vivo* DHEA levels were not affected in our study. While genistein and daidzein do not inhibit SULT2A1, other SULT isoforms, expressed in the liver and kidney which also sulfonate DHEA may be inhibited by these isoflavones, thus potentially accounting for the decreased *in vivo* DHEAS levels that were detected. While SULT2A1 is also abundantly expressed in the liver as well as in the intestine and kidney (35), it has been reported to be induced in the presence of genistein, mediated via the liver X receptor as was shown in the Hep G2 liver cell model (36). These findings suggest that this isoflavone would thus potentially increase DHEAS production. Furthermore, the daidzein sulfoconjugates, daidzein-4′-*O*-sulfate and daidzein-7, 4′-di-*O*-sulfate are potent inhibitors of sterol sulfatase and both conjugates, but not daidzein, have been shown to inhibit the hydrolysis of DHEAS. In addition, neither conjugates nor daidzein have been shown to inhibit sulfotransferase of DHEA (30). These factors thus suggest that isoflavones would rather contribute to the stimulation of DHEAS production and not to the reduction thereof. While genistein and daidzein inhibit steroidogenic enzymes modulating steroidogenesis, the fine balance maintained between the steroid sulfotransferases and sulfatases may be perturbed by the two isoflavonoids that may account for our findings. Genistein and daidzein are both conjugated *in vivo* with SULT1A1, 1A2, 2A1, and 1E1, all capable of sulfonating genistein and daidzein at C4′ and C7 (37), with their sulfonated derivative having been detected in circulation after soy milk dietary supplementation (38). Modulation of DHEAS levels would be dependent on free isoflavone levels as maintained by sulfotransferases and sulfatases activities in steroidogenic tissue, in the liver and kidney, as well as by the competitive inhibition of the aforementioned enzymes by genistein and daidzein. Even though we have shown that free daidzein and genistein levels were detected at 0.07 and 0.34 μM (in females) and 0.125 and 0.43 μM (in males) in circulation, their accumulation in target tissue and intracellular levels together with sulfonated levels, which would determine and contribute to physiological effects, remain unknown and yet to be explored.

These data clearly indicate that while 17,20 lyase activity is significantly inhibited, T and other androgens were largely unaffected. This would suggest that the impact is marginal and therefore ingestion of isoflavones is safe in healthy individuals. However, 17,20 lyase deficiency although rare, is recognized and associated with androgen abnormalities, hypertension, hypokalaemia, and fertility issues and a partial deficiency would theoretically be exacerbated by isoflavone ingestion (39, 40). In addition, it is possible that these isoflavones may interfere with the availability of cholesterol thus impacting steroidogenesis. Daidzein and genistein have been shown to suppress cellular cholesterol biosynthesis significantly in HepG2 cells, while also lowering the cellular esterification of cholesterol. It is possible that these isoflavones may therefore lower the availability cholesterol in the H295R cells potentially contributing to decreased steroid levels in the Δ^5^ pathway since the conversion of cholesterol to PREG is the rate-limiting step in steroidogenesis (41). Basal PREG levels were however very low (≤10 nM). Of interest is a subsequent study which reported that daidzein and genistein both increase mRNA transcripts encoding enzymes involved in cholesterol and lipid metabolism (42).

In conclusion, the *in vivo* clinical data suggest that there was modulation of the Δ5 pathway with potential inhibition of the 17,20 lyase activity within the adrenal in men and within the ovary and adrenal in the females, while the *in vitro* data confirmed that both genistein and daidzein did indeed inhibit the 17,20 lyase activity of CYP17A1, though this is unlikely to impact clinically. In addition, these data confirmed the inhibition of 3βHSD2 by daidzein and genistein, though again this may not be clinically significant in practice. However, taken together, the effects of these isoflavones—the decreased *in vivo* DHEAS and A4 levels and inhibition of the 17,20 lyase activity of CYP17A1, impacts the production of precursor androgens contributing to the pool of active androgens, potentially relevant to clinical conditions associated with androgen excess.

## Ethics Statement

All subjects gave their written informed consent. Both studies received ethical approval by the Research Ethics Committee (East Yorkshire & North Lincolnshire Research Ethics Committee, ref: 09/H1304/45 and 09/1304/45).

## Author Contributions

AS and IJ performed the *in vitro* analysis. TS and SA undertook the clinical trial work. All authors contributed to the final manuscript.

### Conflict of Interest Statement

The authors declare that the research was conducted in the absence of any commercial or financial relationships that could be construed as a potential conflict of interest.
